# Long non-coding RNA TTN antisense RNA 1 facilitates hepatocellular carcinoma progression via regulating miR-139-5p/SPOCK1 axis

**DOI:** 10.1080/21655979.2021.1882133

**Published:** 2021-02-08

**Authors:** Xinghao Zhu, Shiqing Jiang, Zongyao Wu, Tonghua Liu, Wei Zhang, Lili Wu, Lijun Xu, Mingliang Shao

**Affiliations:** a Department of Internal Medicine of Chinese Medicine, Henan University of Chinese Medicine, Zhengzhou, Henan, China; b The First Affiliated Hospital of Henan University of Chinese Medicine, Zhengzhou, Henan, China; c Institute of Tibetan Medicine, Tibet University of Tibetan Medicine, Lhasa, Xizang, China; d Beijing University of Chinese Medicine, Beijing, China; e Institute of Liver Diseases, Shijiazhuang Fifth Hospital, Shijiazhuang, Hebei, China; f Department of Oncology, Shijiazhuang Fifth Hospital, Shijiazhuang, Hebei, China

**Keywords:** TTN-AS1, hepatocellular carcinoma (HCC), MiR-139-5p, SPOCK1, EMT

## Abstract

Reportedly, long non-coding RNAs (lncRNAs) are implicated in hepatocellular carcinoma (HCC) progression, yet little is known concerning the biological functions of TTN antisense RNA 1 (TTN-AS1) in HCC. In this study, quantitative real-time polymerase chain reaction (qRT-PCR) was performed for detecting TTN-AS1, SPOCK1 mRNA, and miR-139-5p expressions in HCC cells and tissues. After TTN-AS1 was overexpressed or knocked down in HCC cells, CCK-8 and 5-Ethynyl-2ʹ-deoxyuridine (EdU) assays were carried out for examining cell multiplication. Transwell assays were conducted for evaluating HCC cell migration and invasion. Dual-luciferase reporter assay was employed for verifying the binding relationships between miR-139-5p and TTN-AS1, and between SPOCK1 3ʹUTR and miR-139-5p. Western blot was employed to measure SPOCK1, E-cadherin, N-cadherin, and Vimentin protein expressions. We demonstrated that, TTN-AS1 and SPOCK1 expression levels were remarkably enhanced in HCC cells and tissues, whereas miR-139-5p expression was observably reduced. Functional experiments suggested that TTN-AS1 knockdown markedly repressed HCC cell multiplication, migration, epithelial-mesenchymal transition (EMT), and invasion. In addition, TTN-AS1 interacted with miR-139-5p and decreased its expression. Moreover, SPOCK1 was a miR-139-5p target, and miR-139-5p inhibitors were able to reverse TTN-AS1 knockdown-induced inhibitory effect on SPOCK1 expression. SPOCK1 overexpression plasmid could counteract TTN-AS1 knockdown-induced inhibiting impact on HCC cell multiplication, migration, invasion, and EMT. In conclusion, TTN-AS1 expression level is remarkably enhanced in HCC, and TTN-AS1 can promote the multiplication, migration, invasion, and EMT of HCC cells via regulating miR-139-5p/SPOCK1 axis.

## Introduction

Hepatocellular carcinoma (HCC) ranks third among the causes of cancer-related deaths [1]. Patients with HCC still carry a poor prognosis due to high recurrence and metastasis rates [2]. Deciphering the molecular mechanism of HCC is essential for developing novel therapeutic approaches.

Long non-coding RNAs (lncRNAs), microRNAs (miRNAs) and other non-coding RNAs (ncRNAs) participate in regulating the tumorigenesis and progression of various malignancies. LncRNAs, characterized by over 200 nucleotides in length, can take part in multiple biological processes, for instance, cell multiplication, metabolism, migration, and apoptosis [3]. Disorder of lncRNAs is associated with many diseases, including cancer [4]. Many lncRNAs, as cancer-promoting factors or tumor suppressors, participate in HCC occurrence and development, for instance, FAL1 [5], MCM3AP-AS1 [6], MALAT1 [7], etc. Reportedly, as an oncogenic lncRNA, lncRNA TTN antisense RNA 1 (TTN-AS1) is implicated in the progression of gastric cancer [8] and esophageal squamous cell carcinoma [9]. Notably, a recent study reports that TTN-AS1 silencing in HCC cells induces apoptosis [10]. Nonetheless, its expression characteristics and biological functions in HCC warrant further investigation.

As a type of ncRNAs with about 18–25 nucleotides in length, miRNAs also partake in regulating diverse biological processes [11]. MiR-139-5p dysregulation is associated with the progression of various cancers [12]. In HCC, miR-139-5p expression is underexpressed, and miR-139-5p can restrain HCC cell multiplication, migration and invasion [13]. The mechanism of its dysregulation in HCC is, however, still unclear. LncRNAs can sponge miRNAs by serving as competitive endogenous RNAs (ceRNAs), by which they participate in tumorigenesis and cancer progression [14]. TTN-AS1 facilitates the development of breast cancer by modulating miR-139-5p/ZEB1 axis [15]. Another study reports that, TTN-AS1 promotes ovarian cancer cell multiplication, colony formation, migration, and invasion via regulating miR-139-5p/ROCK2 axis [16]. Whether TTN-AS1 could adsorb miR-139-5p in HCC cells remains to be investigated.

Sparc/osteonectin, cwcv, and kazal-like domains proteoglycan 1 (SPOCK1) is a gene encoding a seminal plasma proteoglycan. As an oncogene, it regulates cell cycle, proliferation, and apoptosis, thus participating in the development of multiple tumors, including gastric cancer [17], colorectal carcinoma [18], prostate carcinoma [19], and so on. Besides, there is evidence that SPOCK1 expression is markedly increased in HCC, and SPOCK1 can inhibit apoptosis and promote migration and invasion of HCC cells [20]. Nevertheless, the mechanism of its dysregulation in HCC needs to be clarified.

The aim of the present research is to explore the expression characteristics, biological functions, and underlying mechanism of TTN-AS1 in HCC. We demonstrated that TTN-AS1 expression was significantly elevated in HCC, and TTN-AS1 promoted the malignant biological behaviors of HCC cells via directly targeting miR-139-5p to modulate SPOCK1 expression. The present study offered potential targets for HCC therapy.

## Materials and methods

### Tissue samples

Cancerous and paracancerous tissues were surgically removed from 70 HCC patients who were enrolled at the First Affiliated Hospital of Henan University of Chinese Medicine, and then immediately stored in liquid nitrogen. None of the subjects underwent radiotherapy or chemotherapy prior to the surgery. The study got the approval of the Ethics Committee of the First Affiliated Hospital of Henan University of Chinese Medicine, and all of the subjects signed informed consent.

### Cell culture

Normal human hepatocyte cell line L02 and the four kinds of HCC cell lines (BEL-7402, Hep3B, HepG2, and Huh7) were obtained from American Type Culture Collection (ATCC; Manassas, VA, USA). These cells were cultured in Dulbecco’s Modified Eagle’s Medium (DMEM; Invitrogen, Carlsbad, CA, USA) containing 0.1 mg/ml streptomycin, 100 U/ml penicillin (Hyclone, Logan, UT, USA), and 10% fetal bovine serum (FBS; Gibco, Carlsbad, CA, USA) at 37°C in 5% CO_2_. The cells during logarithmic growth were collected for the follow-up experiments.

### Cell transfection

Lipofectamine® 3000 (Invitrogen, Carlsbad, CA, USA) was adopted to conduct cell transfection. Two short hairpin RNAs (shRNAs) targeting TTN-AS1 (sh-TTN-AS1-1 and sh-TTN-AS1-2) and shRNA control (sh-NC) were obtained from GenePharma (D010003; Shanghai, China). MiR-139-5p mimics, miR-139-5p inhibitors, and the negative control miRNAs (miR-NC) were obtained from RiboBio (Guangzhou, China). SPOCK overexpression plasmid and empty plasmid were bought from GenePharma (LV-4; Shanghai, China).

### Quantitative real-time polymerase chain reaction (qRT-PCR)

TRIzol reagent (Invitrogen, Carlsbad, CA, USA) was utilized for total RNA extraction from HCC cell lines and tissues. RNA was reversely transcribed into cDNA using the RevertAid First Strand cDNA Synthesis Kit (Thermo-Fisher Scientific, Waltham, MA, USA). Subsequently, with cDNA as the template, SYBR Premix Ex Taq™ (TaKaRa, Otsu, Shiga, Japan) was utilized for qRT-PCR. Ultimately, the calculation of the relative expressions of the genes was performed with 2^−ΔΔCt^ method. U6 and GAPDH acted as the internal references. Below were the primer sequences (‘F’ represents ‘forward’; ‘R’ represents ‘reverse’): TTN-AS1, 5ʹ-GCCAGGTAGAGTTGCAGGTT-3ʹ (F) and 5ʹ-GAAGCTGCTGCGGATGAATG-3ʹ (R); miR-139-5p, 5ʹ-TCTACAGTGCACGTGTC-3ʹ (F) and 5ʹ-TGGTGTCGTGGAGTCG-3ʹ (R); SPOCK1, 5ʹ-CAGCCTGTCCACACAAAAGC-3ʹ (F) and 5ʹ-CCATCGATTTGGGGGTTCCA-3ʹ (R); GAPDH, 5ʹ-CAGGAGGCATTGCTGATGAT-3ʹ (F) and 5ʹ-GAAGGCTGGGGCTCATTT-3ʹ (R); U6, 5ʹ-CTCGCTTCGGCAGCACA-3ʹ (F) and 5ʹ-AACGCTTCACGAATTTGCGT-3ʹ (R).

### Cell counting kit-8 (CCK-8) assay

The HCC cells were transferred into 96-well plates (1 × 10^3^ cells/well), and then each well was added with 10 µL of CCK-8 solution (Sigma-Aldrich, St. Louis, Mo, USA) at 0, 24, 36, and 72 h, with which the cells were incubated for 2 h. Then a microplate reader (Thermo-Fisher Scientific, Waltham, MA, USA) was employed for measuring the absorbance of the cells at 450 nm. 72 h later, the proliferation curve of the cells was plotted based on the values of absorbance.

### 5-Ethynyl-2ʹ-deoxyuridine (EdU) assay

EdU kit (RiboBio, Guangzhou, China) was adopted for EdU assay. Cells were inoculated into 96-well plates, followed by adding EdU solution (50 μmol/L) to each well. After 2 h of incubation, the cells were rinsed twice with PBS, fixed with 4% paraformaldehyde for 30 min, and incubated with glycine for 10 min. Then 0.5% TritonX-100 was used to incubate the cells. Next, Apollo staining solution was used for staining the cells for 30 min at room temperature in the dark, and the cell nuclei were then stained by DAPI staining solution for 30 min. Ultimately, a ﬂuorescence microscope (Nikon Eclipse Ti Microscope, Tokyo, Japan) was utilized for detecting the percentage of EdU-positive cells.

### Transwell assay

HCC cell migration and invasion capabilities were examined by 24-well Transwell chambers (8 µm pore size; Corning, NY, USA). In migration assay, the top compartment containing 200 μL of serum-free medium was added with 4 × 10^4^ HCC cells. Meanwhile, the bottom compartment was added with 600 μL of medium with 10% FBS. After being cultured for 48 h, cotton swabs were used to remove the cells remaining in the upper compartment, and the cells passing through the filter were fixed with 4% paraformaldehyde and stained with 0.4% crystal violet solution. Subsequently, under a microscope (Olympus, Tokyo, Japan), the migrated cells were counted. In the invasion assay, before the cells were inoculated, the filter was coated with a layer of Matrigel (BD, Bedford, MA, USA), and the other procedures were the same as the migration assay.

### Western blot

RIPA lysis buffer (Beyotime, Shanghai, China) was employed for lysing HCC cells to extract the total protein, and BCA Protein Assay (Thermo-Fisher Scientific, Waltham, MA, USA) was utilized for quantifying the protein in the samples. Subsequently, 20 μg protein in each group was separated using SDS-PAGE. Following the electrophoresis, proteins were transferred onto polyvinylidene difluoride (PVDF) membranes (Millipore, Billerica, MA, USA). Next, the membranes and primary antibodies (anti-SPOCK1: 1:2000, ab229935, Abcam, USA; anti-E-cadherin: 1:2000, ab233611, Abcam, USA; anti-Vimentin: 1:2000, ab92547, Abcam, USA; anti-N-cadherin: 1:2000, ab254512, Abcam, USA; anti-GAPDH: 1:5000, ab9485, Abcam, USA) were incubated overnight at 4°C, and then the membranes and secondary antibody (goat anti-rabbit IgG-HRP, 1:10000, ab6721; Abcam, USA) were incubated for 1 h at room temperature. Ultimately, enhanced chemiluminescence (ECL) substrate kit (Amersham Biosciences, Little Chalfont, UK) was employed to show the protein bands, and GAPDH acted as the internal reference.

### Dual-luciferase reporter assay

Wild type (wt) and mutated type (mut) luciferase reporter vectors, including pMIR-REPORT-SPOCK1-3ʹUTR-wt/mut and pmiR-REPORT-TTN-AS1-wt/mut, were constructed by Genecreate (Wuhan, China). Subsequently, HEK-293 T cells were transfected with luciferase reporters and miR-139-5p mimics or miR-NC, respectively. After 48 h, the luciferase activity of the cells in each group was measured using the Dual-Luciferase Reporter Assay System (Promega, Madison, WI, USA).

### Statistical analysis

All data were expressed as mean ± standard deviation, and SPSS 19.0 software (SPSS Inc., Chicago, IL, USA) was the statistical analysis tool. Student’s *t*-test and one-way ANOVA test were carried out for analyzing the difference between the groups, and Chi-square test was conducted for examining the correlation between TTN-AS1 expression and HCC patients’ clinicopathological characteristics. Pearson’s correlation coefficient was used for determining the correlations among TTN-AS1, miR-139-5p, and SPOCK1 mRNA. *P* < 0.05 denoted statistical significance. The experiments herein were conducted at least in triplicate.

## Results

### TTN-AS1 expression was notably elevated in HCC cell lines and tissues

**Figure 1.**
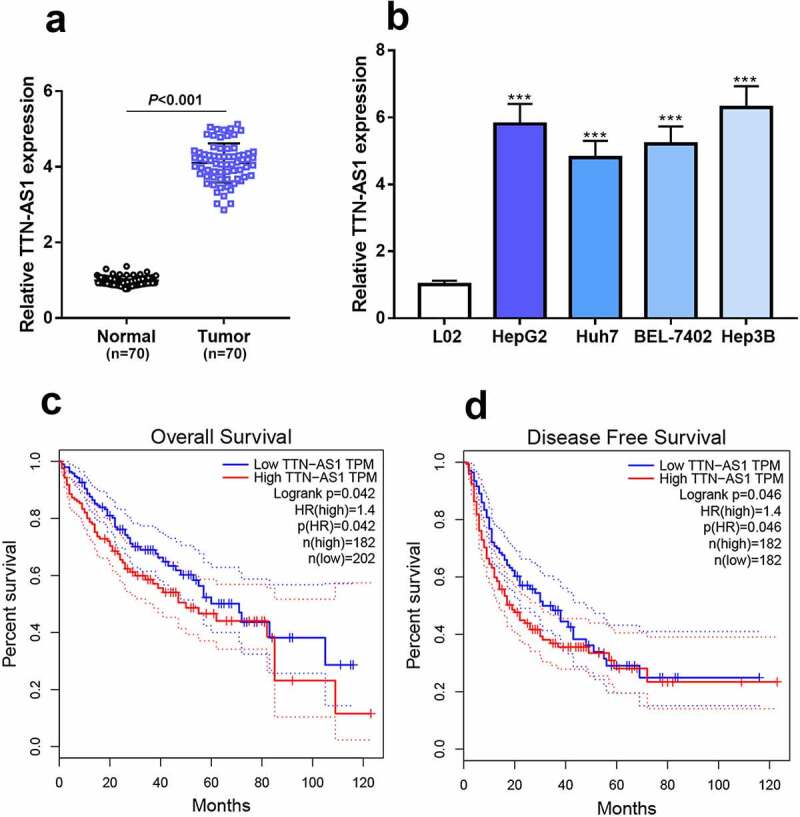
TTN-AS1 expression was significantly up-regulated in HCC tissues and cell lines

TTN-AS1 is reported to be up-regulated in multiple cancers [8,9,21,22]. To clarify the characteristics of TTN-AS1 expression in HCC tissues and cell lines, qRT-PCR was conducted and it was shown that TTN-AS1 expression in HCC tissues was observably higher as opposed to paracancerous tissues (Figure 1(a)). Consistently, in HCC cell lines, TTN-AS1 expression was markedly higher, compared with that in the L02 cell line (Figure 1(b)). Moreover, after the patients were divided into TTN-AS1 high expression group and low expression group, chi-square test showed that, highly expressed TTN-AS1 was associated with advanced TNM stage and larger tumor size (Table 1). Additionally, GEPIA online database manifested that high TTN-AS1 expression was related to shorter overall survival time and disease-free survival time of the patients (Figure 1(c,d)). The aforementioned evidence suggested that TTN-AS1 probably participated in HCC progression.

**Table 1.** Relationship between TTN-AS1 expression and clinicopathological features

		TTN-AS1		
Features	n	High (n = 35)	Low (n = 35)	*P*
Age (years)				
≥ 60	38	16	22	0.230
< 60	32	19	13	
Gender				
Male	26	13	13	0.804
Female	44	22	12	
T stage				
T1-T2	41	27	14	0.003
T3-T4	29	27	21	
N stage				
N0	30	20	10	0.029
N1-2	40	6	20	
M stage				
M0	43	27	16	0.014
M1	27	19	27	
Tumor size (cm)				
≥ 5	41	26	15	0.015
< 5	29	9	20

(a-b) qRT-PCR showed that TTN-AS1 expression was up-regulated in HCC tissues (a) and cell lines (b).(c-d) GEPIA database showed high TTN-AS1 expression was related to shorter overall survival time (c) and disease-free survival time (d).Student’s *t*-test and one-way ANOVA test, *** *P* < 0.001.

### TTN-AS1 knockdown repressed the malignant biological behaviors of HCC cells

**Figure 2.**
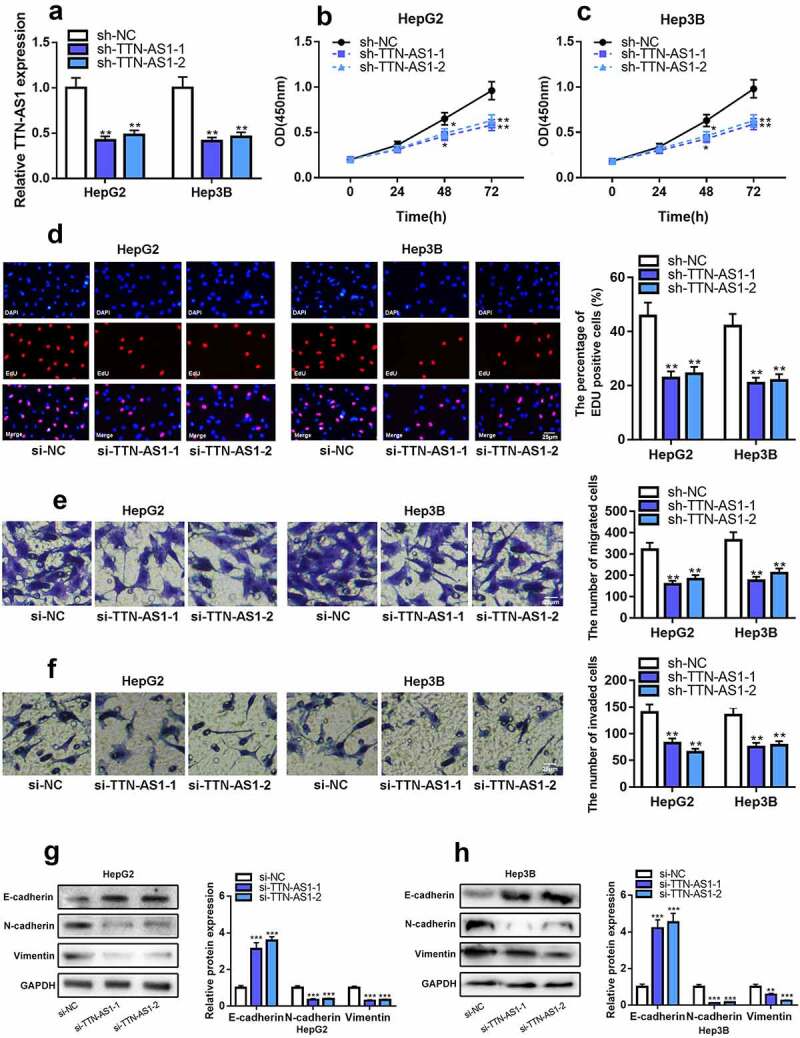
TTN-AS1 knockdown could inhibit the proliferation, migration, invasion, and EMT of HCC cells

Reportedly, TTN-AS1 is involved in the progression of various types of cancer as an oncogenic lncRNA [8,9,21,22]. In HCC, TTN-AS1 knockdown could induce the apoptosis of cancer cells [10]. To pinpoint the regulatory effects of TTN-AS1 on HCC cells’ multiplication, migration, invasion, and epithelial-mesenchymal transition (EMT), two shRNAs were used to knock down TTN-AS1 expression in HepG2 and Hep3B cells, respectively, and, as shown, both two shRNAs had high knockdown efficiency (Figure 2(a)). EdU and CCK-8 assays evinced that in comparison to the control group, knocking down TTN-AS1 could significantly inhibit the proliferation of HepG2 and Hep3B cells (Figure 2(b–d)). Transwell assay suggested that in comparison to those in si-NC group, cell migration and invasion capacities were significantly reduced in the si-TTN-AS1-1 and si-TTN-AS1-2 groups (Figure 2(e,f)). Moreover, the expression level of E-cadherin was elevated, and the expression levels of Vimentin and N-cadherin in HepG2 and Hep3B cells were decreased after the transfection of TTN-AS1 shRNAs (Figure 2(g,h)). The above-mentioned evidence confirmed that knocking down TTN-AS1 could remarkably suppress the malignant phenotypes of HCC cells.

(a) qRT-PCR showed that TTN-AS1 expression in HCC cells was decreased after the transfection of TTN-AS1 shRNAs.(b-c) CCK-8 assay showed that HCC cell proliferation was decreased after the transfection of TTN-AS1 shRNAs.(d) EdU assay showed that HCC cell proliferation was inhibited after the transfection of TTN-AS1 shRNAs.(e-f) Transwell assay showed that HCC cell migration and invasion were repressed after the transfection of TTN-AS1 shRNAs.(G-H) Western blot assay showed that the expression level of E-cadherin was increased, and expression levels of N-cadherin and Vimentin were decreased in HepG2 and Hep3B cells after the transfection with TTN-AS1 shRNA. Student’s *t*-test, * *P* < 0.05, ** *P* < 0.01, *** *P* < 0.001.

### TTN-AS1 regulated SPOCK1 expression by sponging miR-139-5p

**Figure 3.**
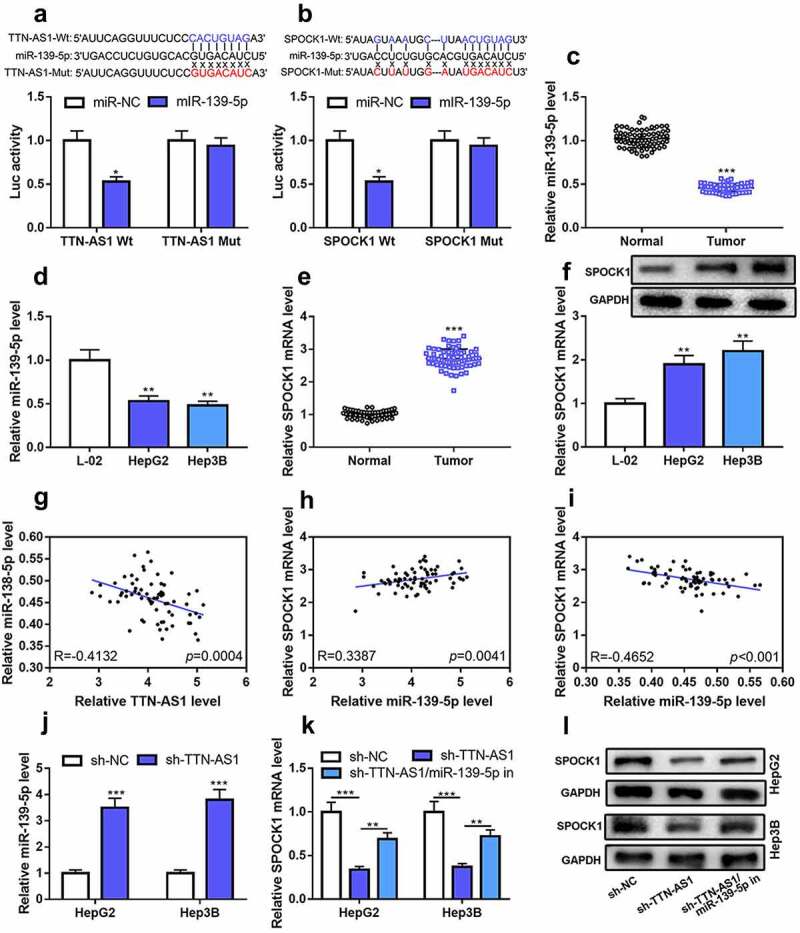
TTN-AS1 adsorbed miR-139-5p as a sponge to regulate the expression of SPOCK1

To delve into the mechanism by which TTN-AS1 played a role in HCC progression, StarBase database (http://starbase.sysu.edu.cn/) was searched, and it showed that the SPOCK1 3ʹUTR and TTN-AS1 both contained the binding site complementary to miR-139-5p. Dual-luciferase reporter assay elucidated that miR-139-5p mimics reduced TTN-AS1-WT reporter’s luciferase activity, yet failed to affect TTN-AS1-MUT vector’s luciferase activity (Figure 3(a)), and these results suggested that miR-139-5p was a target of TTN-AS1, which was consistent with the previous studies [15,16]. Similarly, the co-transfection of miR-139-5p reduced SPOCK1-WT reporter’s luciferase activity, but could not alter that of SPOCK1-MUT reporter (Figure 3(b)). It was also discovered that miR-139-5p expression was markedly reduced, whereas SPOCK1 expression was observably enhanced in HCC tissues and cell lines (Figure 3(c–f)). Pearson’s correlation analysis showed that miR-139-5p and TTN-AS1 expressions in HCC tissues were negatively correlated, and SPOCK1 mRNA and TTN-AS1 expressions were positively correlated, and SPOCK1 mRNA and miR-139-5p expressions were inversely related (Figure 3(g–i)). In addition, we found that knocking down TTN-AS1 in HepG2 and Hep3B could significantly up-regulate miR-139-5p expression and suppress SPOCK1 expression; the co-transfection of miR-139-5p inhibitors in HepG2 and Hep3B cells counteracted TTN-AS1 knockdown-induced inhibiting effect on SPOCK1 expression level (Figure 3(j–l)). Collectively, TTN-AS1 could probably adsorb miR-139-5p as a molecular sponge to up-regulate SPOCK1 expression in HCC.

(a-b) Luciferase reporter assay validated the binding sites between TTN-AS1 and miR-139-5p as well as between miR-139-5p and SPOCK1 3ʹUTR. (c-d) qRT-PCR showed that miR-139-5p expression in HCC tissues and cell lines was decreased. (e-f) qRT-PCR and Western blot assay showed that SPOCK1 mRNA and protein expression levels in HCC tissues and cell lines were increased. (g-i) Pearson’s correlation analysis showed that, in HCC samples, miR-139-5p expression was negatively correlated with TTN-AS1 expression and SPOCK1 mRNA expression, and TTN-AS1 expression was positively correlated with SPOCK1 mRNA expression. (j) qRT-PCR showed that the expression of miR-139-5p was increased in HCC cells after transfection of TTN-AS1 shRNA.(k-l) qRT-PCR and Western blot showed that SPOCK1 mRNA and protein expression levels were decreased after the transfection of TTN-AS1 shRNA, but these effects were reversed by the co-transfection of miR-139-5p inhibitor. Student’s *t*-test, one-way ANOVA test, * *P* < 0.05, ** *P* < 0.01 and *** *P* < 0.001.

### Overexpression of SPOCK1 could abolish TTN-AS1 knockdown-induced effects on the malignant biological behaviors of HCC cells

**Figure 4.**
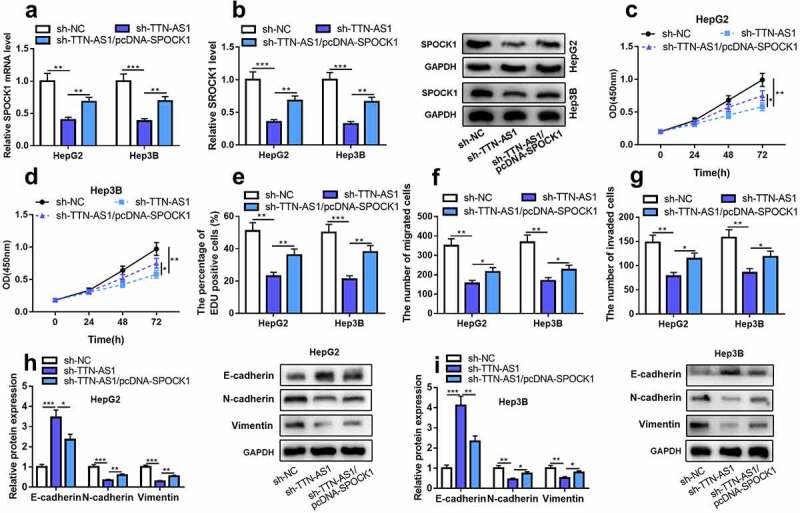
Overexpression of SPOCK1 could reverse the effects of knocking down TTN-AS1 on the proliferation, migration, invasion, and EMT of HCC cells

Subsequently, we further investigated whether TTN-AS1 could modulate the malignant phenotypes of HCC cells in a SPOCK1-dependent manner. It was shown that SPOCK1 overexpression plasmid could significantly reverse TTN-AS1 knockdown-induced inhibitory effects on SPOCK1 expression (Figure 4(a,b)). Subsequently, with CCK-8 assay, EdU assay and Transwell assay, it was discovered that SPOCK1 restoration significantly abolished TTN-AS1 knockdown-caused inhibiting effects on HCC cell multiplication, migration, and invasion (Figure 4(c–g)). Additionally, we detected the expression levels of the EMT markers. As shown, TTN-AS1 knockdown promoted the expression of E-cadherin, but repressed the expression of N-cadherin and Vimentin; the co-transfection of SPOCK1 overexpression plasmid reversed these changes (Figure 4(h,i)). The above-mentioned findings demonstrated that TTN-AS1 facilitated the malignant phenotypes of HCC cells via modulating SPOCK1.

(a-b) qRT-PCR showed that the expressions of SPOCK1 mRNA and protein in HCC cells were suppressed after the transfection of TTN-AS1 shRNA, and these effects were reversed after the co-transfection of SPOCK1 overexpression plasmids. (c-d) CCK-8 assay showed that HCC cell proliferation was repressed after the transfection of TTN-AS1 shRNA, which was counteracted by SPOCK1 overexpression plasmids. (e) EdU assay showed that HCC cell proliferation was suppressed after the transfection of TTN-AS1 shRNA, which was counteracted by SPOCK1 overexpression plasmids. (f-g) Transwell assay showed that cell migration and invasion were inhibited after the transfection of TTN-AS1 shRNA, which were counteracted by SPOCK1 overexpression plasmids. (h-i) Western blot indicated that, in HCC cells, TTN-AS1 knockdown promoted E-cadherin expression, and inhibited N-cadherin expression and Vimentin expression; however, these effects could be reversed by SPOCK1 restoration. Student’s *t*-test, * *P* < 0.05, ** *P* < 0.01, and *** *P* < 0.001.

## Discussion

LncRNAs can regulate gene expression through different mechanisms to participate in tumor progression, such as, chromatin modification, transcriptional regulation, and post-transcriptional regulation [23]. Environmental factors (such as smoking), virus infection, genomic alteration, and other factors may cause the dysregulation of lncRNA in cancer [24,25]. The dysregulation of TTN-AS1 is implicated in the tumorigenesis and progression of many tumors. For example, in osteosarcoma, TTN-AS1 expression is markedly enhanced, and high TTN-AS1 expression is associated with the patients’ poor prognosis, and TTN-AS1 facilitates cancer cell multiplication and suppresses apoptosis via regulating the miR-134-5p/MBTD1 axis [21]. In lung adenocarcinoma, TTN-AS1 expression is also remarkably up-regulated and TTN-AS1 can sponge miR-142-5p to increase CDK5 expression, thus facilitating cell migration, invasion and EMT [22]. In gastric carcinoma, TTN-AS1 is highly expressed, and it serves as a ceRNA, sponging miR-376b-3p, to promote the malignant phenotypes of gastric cancer cells [8]. The silencing of TTN-AS1 in HCC cells induces apoptosis through regulating PTEN/Akt signal pathway [10]. Herein, our work authenticated that TTN-AS1 expression was markedly elevated in HCC tissues and cells, and high TTN-AS1 expression was connected with advanced TNM stage and larger tumor size. In addition, knocking down TTN-AS1 markedly restrained HCC cell multiplication, migration, and invasion. Our research also displayed that TTN-AS1 functioned as an oncogenic lncRNA in HCC.

According to ceRNA theory, lncRNAs can act as molecular sponges to decoy miRNAs and thus modulate the expression levels of downstream genes. To decipher TTN-AS1’s molecular mechanism in HCC, in this work, a ceRNA network consisting of TTN-AS1, miR-139-5p, and SPOCK1 was established. MiR-139-5p contributes to suppressing the progression of many tumors. For example, the expression of miR-139-5p is observably reduced in endometrial carcinoma, and miR-139-5p can target HOXA10 to restrain cancer cell multiplication and migration [26]. MiR-139-5p expression is also markedly down-regulated in gallbladder carcinoma, and miR-139-5p represses the malignant biological behaviors of gallbladder carcinoma cells via targeting PKM2 [27]. In HCC, miR-139-5p expression is notably reduced, which is modulated by SNHG3, and miR-139-5p targets BMI1 to repress HCC cell multiplication, migration, and invasion [13]. Another study reports that miR-139-5p represses HCC cell migration, invasion and EMT by targeting ZEB1 and ZEB2 [28]. The current study revealed that miR-139-5p expression level in HCC tissues and cells was notably lower as against in paracancerous tissues and normal human liver cell lines, which was consistent with the previous reports [13,28]. Moreover, it was unmasked that SPOCK1 was a target gene of miR-139-5p, and TTN-AS1 had a regulatory effect on miR-139-5p. Our study not only partly clarified the mechanism of miR-139-5p dysregulation, but also depicted miR-139-5p’s downstream mechanism in HCC progression.

**Figure 5.**
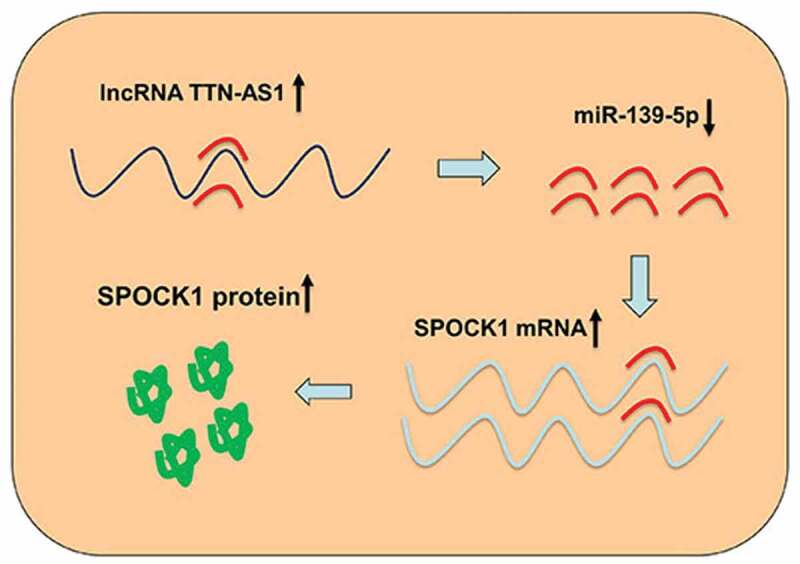
Graphic abstract

SPOCK1 is originally found in human seminal plasma, belonging to the family of secreted protein acidic and rich in cysteine (SPARC) [29]. The SPARC family members share a similar structure, consisting of N-terminus, C-terminus, and follistatin-like domain [30]. SPOCK1 can participate in embryonic development, tissue remodeling, and other physiological processes [31]. In addition, it can regulate the growth, metastasis, and drug resistance in a variety of cancers [31]. In HCC, reportedly, SPOCK1 expression is significantly increased in about 60% of HCC samples, and high SPOCK1 expression is associated with the HCC patients’ poor prognosis [20]. SPOCK1 can be modulated by CHD1L, and elevated SPOCK1 expression facilitates HCC tumorigenesis in mice by activating Akt signaling and represses HCC cell apoptosis via regulating the release of cytochrome c and activities of caspase-3 and caspase-9 [20]. In HCC, miR-193a-5p, miR-139-5p, and miR-940 can target SPOCK1 to inhibit HCC progression [32]. Consistently, the present study demonstrated that SPOCK1 expression was notably up-regulated in HCC. Additionally, SPOCK1 mRNA expression in HCC tissues was inversely related to miR-139-5p expression and positively correlated with TTN-AS1 expression. MiR-139-5p inhibitors counteracted TTN-AS1 knockdown-induced inhibitory effects on SPOCK1 expression, and overexpression of SPOCK1 could counteract TTN-AS1 knockdown-caused inhibiting effects on HCC cell malignant phenotypes. Therefore, it was confirmed that TTN-AS1 could regulate HCC development via miR-139-5p/SPOCK1 axis (Figure 5).

LncRNA TTN-AS1 is a ceRNA for miR-139-5p, and it can up-regulate SPOCK1 to facilitate HCC progression.

## Conclusion

This work indicates that TTN-AS1 is markedly up-regulated in HCC, and TTN-AS1 facilitates HCC progression via increasing SPOCK1 expression and repressing miR-139-5p expression. The present study helps clarify HCC progression’s molecular mechanism and provides potential therapy targets for this deadly disease.

## Highlights

- TTN-AS1 expression was notably elevated in HCC cell lines and tissues
- TTN-AS1 knockdown suppressed HCC cell multiplication, migration, and invasion

TTN-AS1 regulated SPOCK1 expression by sponging miR-139-5p
